# The Management of Labor and the Lying-in Period

**Published:** 1885-11

**Authors:** 


THE MANAGEMENT OF LABOR AND OF THE LYING-IN PERIOD. A guide for the young practitioner. By Henry G. Landis, A. M., M. D., Professor of Obstetrics and Diseases of Women in Starling Medical College, Fellow of the American Academy of Medicine; member of the American Medical Association; author of "How to Use the Forceps;" "a Compend of Obstetrics," etc., etc. Philadelphia: Lea Brothers & Co. 1885.

The aim of this book, as the author informs us, is to serve as a guide to practice, divested of all superfluous or irrelevant details. It takes for granted an acquaintance with the anatomy and physiology of the parts involved, and of the mechanism of natural labor, alluding to such subjects only when a right understanding of them is necessary to explain and justify the course recommended.

The paper, printing and illustrations are in the best style of this reputable house.

